# Author Correction: Malaria trends in Ethiopian highlands track the 2000 ‘slowdown’ in global warming

**DOI:** 10.1038/s41467-026-69644-1

**Published:** 2026-02-13

**Authors:** Xavier Rodó, Pamela P. Martinez, Amir Siraj, Mercedes Pascual

**Affiliations:** 1 ICREA and CLIMA (Climate and Health) Program, ISGlobal, Barcelona, Spain; 2 Department of Epidemiology, Center for Communicable Disease Dynamics, T.H. Chan School of Public Health, Harvard University, Boston, MA USA; 3 Department of Biological Sciences, University of Notre Dame, Notre Dame, IN USA; 4 Department of Ecology and Evolution, University of Chicago, Chicago, IL USA; 5 Santa Fe Institute, Santa Fe, NM USA; 6 Present Address: Department of Microbiology and Department of Statistics, University of Illinois at Urbana, Champaign, Champaign, IL USA

Correction to: *Nature Communications* 10.1038/s41467-021-21815-y, published online 10 March 2021.

In the version of this article initially published, while the code for the transmission rate *β*, equation (8), was correctly implemented in the code for the numerical simulation of the model and its fitting to the time series data, as shared in Github, the final term “+ *b*_*T*_ TEMP” and associated description was missing from the equation (8), which has now been corrected to read

$$\beta \left(t\right)=\exp \left[{\sum }_{k=1}^{6}{b}_{k}{s}_{k}+{b}_{{T}_{4}}{S}_{4}{{{{\rm{TEMP}}}}}_{1}+{b}_{{T}_{6}}{S}_{6}{{{{\rm{TEMP}}}}}_{2}+{b}_{T}{{{\rm{TEMP}}}}\right]\frac{dT}{dt}$$

in the HTML and PDF versions of the article.

