# Supplementary material for: Malaria trends in Ethiopian highlands track the 2000 ‘slowdown’ in global warming
Source: Nat Commun. 2021 Mar 10;12:1555. doi: 10.1038/s41467-021-21815-y (PMC7946882; doi:10.1038/s41467-021-21815-y)
Supplement: Supplementary file 1 — Supplementary Information [file 41467_2021_21815_MOESM1_ESM.pdf]

Supplementary Information

Malaria trends in Ethiopian highlands track the 2000 'slowdown' in global warming

Xavier Rodo[1], Pamela P. Martinez[2], Amir Siraj[3], Mercedes Pascual[4*].

Supplementary Note 1: Further information on the regional climate database

Figure 1a shows the location of the 24 weather stations for Oromia (Ethiopia) from January 1993 through December 2007. For the interval 1968-2008, the 11 closest stations to DZ and the study area used in the construction of the DZ_{reg} series were: Abomsa, Fiche, Kulumsa, Langano, Mendida, Metehara (NMSA), Mojo, Robe, Ziway, Adama and Assela. The DZ_{reg} temperature time series were assessed for their similarities with: (1) the composited Debre Zeit (DZ) 4-station average from the previous study of global warming and malaria[7], and (2) the average of the 24 series in Oromia (ORO). Correlation values for the inter-comparison between DZ and DZ_{reg} (the nearest 11 stations to DZ), show high consistency (r_{xy}=0.92; *p<0,002*). This is also the case between DZ and the average of the 24 stations in the region (ORO), albeit lower in the latter (r_{xy}=0.79; *p<0,01*)

Supplementary Figures

Supplementary Figure 1: Comparison of minimum temperature time series. Temporal coevolution of T_{min} between DZ and the 24 weather stations in Oromia for the interval 1993-2008 (ORO in Supp. Fig. 2a), and for the generated DZ_{reg} dataset 1968-2008. A) T_{min} is shown for the 24 stations and also for two averages of the set, obtained respectively by excluding the DZ station (thick black line) and by including it (thick red line). Note the effect of the movement up in elevation of the DZ station after 2004, reflected by a step change to a lower mean. The 24-ensemble average retains however the interannual structure of DZ (e.g. there is a large dip coincident with the 2006 EN event). B) T_{min} is shown for the average (blue, referred to as DZ_{reg}) of the 11 series closest to the DZ station (see Methods). Note here too the step decrease of the mean reflecting the change in location of the DZ station, and how the interannual variability of this station is otherwise closely mimicked by the composite one (including the large concomitant dip coincident with the 2006 EN event).

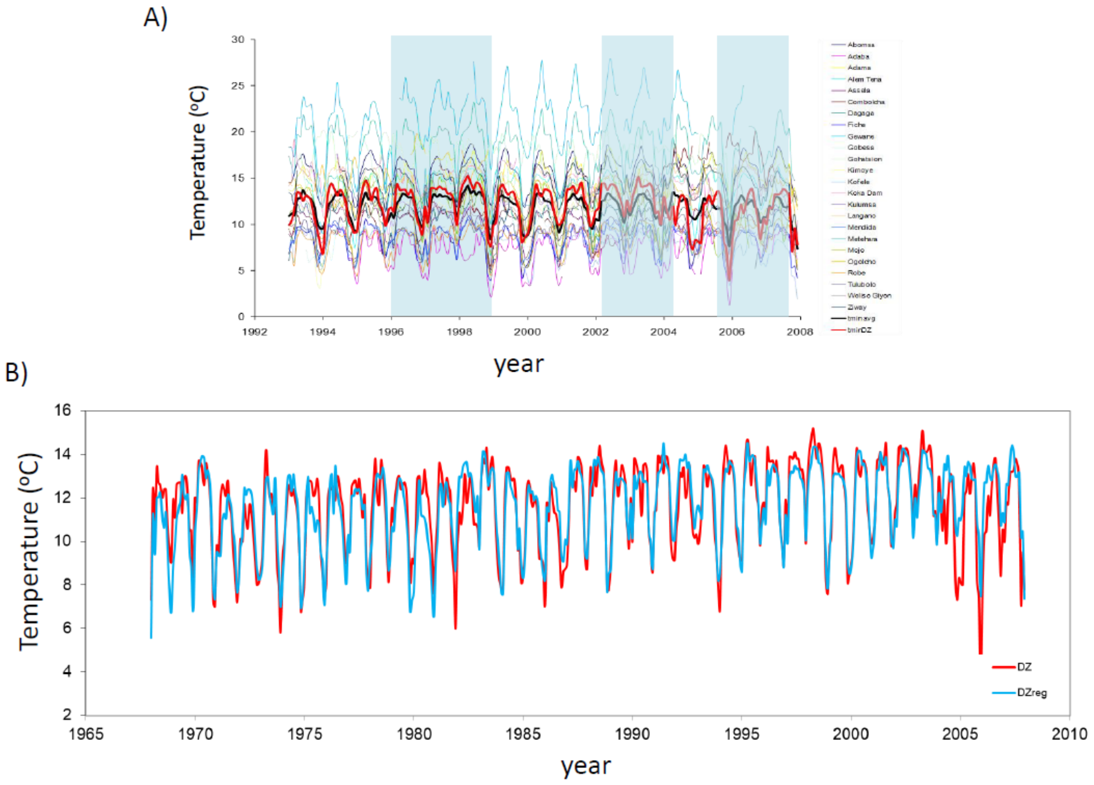

Supplementary Figure 2: Comparison of the composite time series for T_{max} with and without including the DZ station. A) Average of the 24 stations within Oromia with (ORO+DZ; blue) and without (ORO-DZ, yellow) DZ. B) Same as for A) but for the DZ_{reg} series (DZ_{reg}+DZ and DZ_{reg}-DZ; DZ_{reg}, the average of the 11 stations closest to DZ).

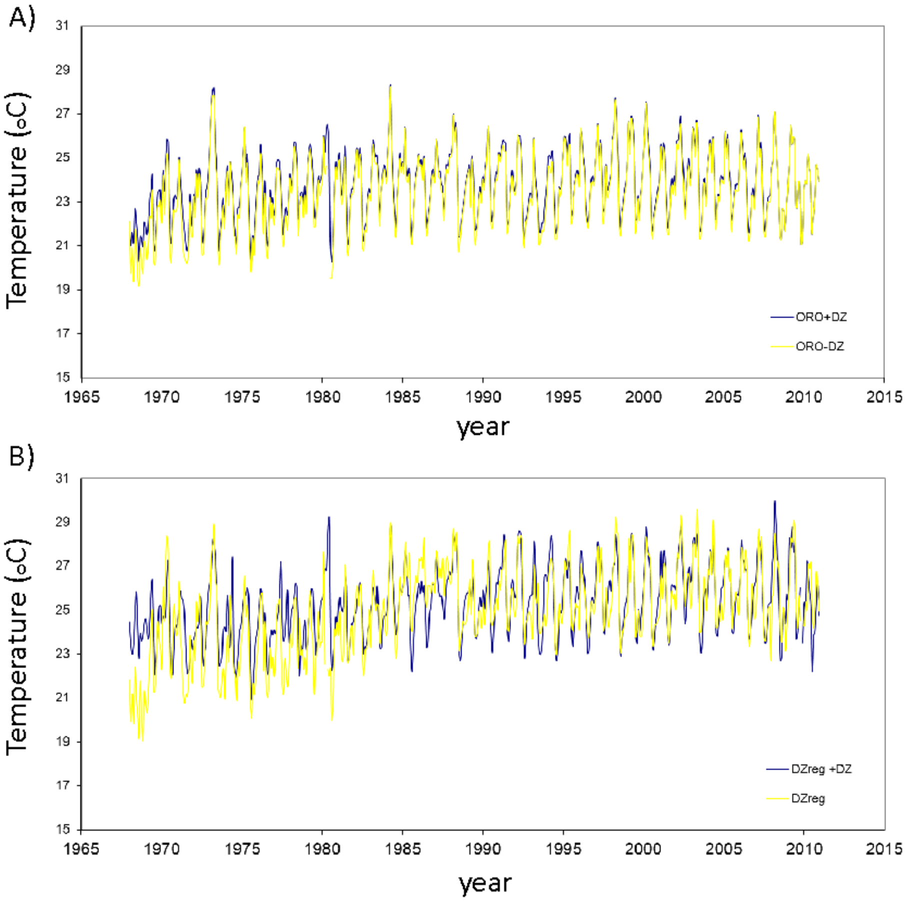

Supplementary Figure 3: Profile likelihood curves for estimated parameters of the transmission model, with corresponding Maximum Likelihood Estimates (MLE) and confidence intervals. Top graphs show the Profile Likelihood Curves for estimated parameters, from which confidence intervals are obtained (as the points at which the profile curve crosses the horizontal line two log-likelihood units below the MLE). The MLE and confidence interval of each parameter is provided in the table. The MLE estimates for the temperature coefficients b_{T4} and b_{T6} are positive indicating a significant effect of the climate covariate that increases transmission rates during the respective seasonal windows of time with warming. Most parameters exhibit reasonable estimates with well-defined CIs. The role of class Q is to add a reservoir of transmission via an asymptomatic class; its estimated duration can become arbitrarily low. The estimated rate at which infected individuals flow into this class is much lower than that at which they return to S. As the number of individuals in Q is therefore small, its effect on temporal dynamics of cases is negligible and the model fit is unable to constrain the lower limit of the length that individuals spend in this class. This is consistent with an asymptomatic class not playing an important role in regions of unstable transmission. The estimated length of the incubation period is lower than empirical values. In the model, the effective delay between seasonal forcing of transmission and incidence/peak cases is an emergent value depending on several processes: seasonality, parasite development within the mosquito (implicitly, through parameter τ), and the incubation period in humans. Thus, we expect the estimated incubation period to establish that delay as best as possible given model structure, and not necessarily to match values of this parameter interpreted solely as an incubation period. The shape of the estimated seasonal transmission rate and its interannual variation due to temperature covariates and parameters bT4 and bT6 are shown in Suppl. Fig. 6.

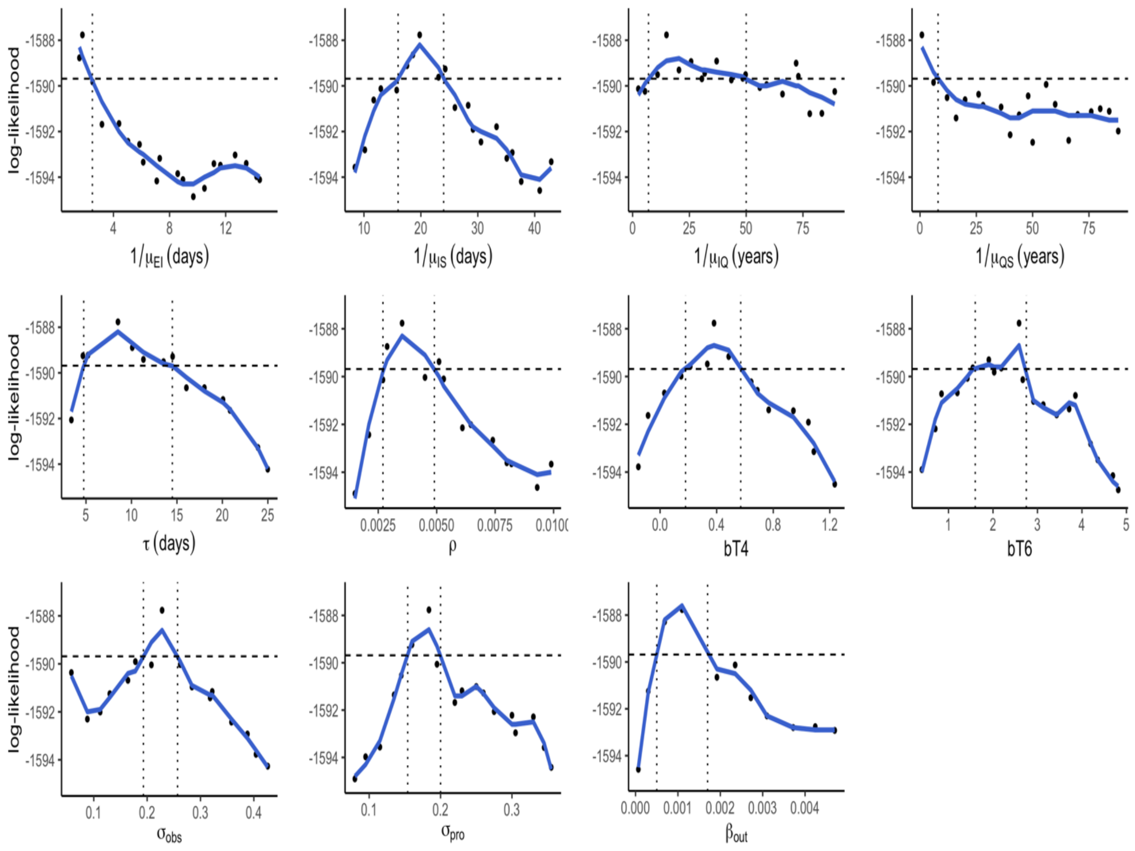

Parameter	Value	Confidence Interval
**1/muEI**	1.8 (days)	0.0, 2.5 (days)
**1/muIS**	20 (days)	16, 24 (days)
**1/muIQ**	15 (years)	7, 50 (years)
**1/muQS**	0.8 (years)	0, 8 (years)
**tau**	8.5 (days)	4.8, 14.5 (days)
**rho**	0.0035	0.0027, 0.0049
**bT4**	0.38	0.18, 0.57
**bT6**	2.59	1.60, 2.75
**betaOUT**	0.0011	0.0005, 0.0017
**sigma OBS**	0.23	0.19, 0.26
**sigma PRO**	0.18	0.15, 0.20

Supplementary Figure 4: Exploration of the functional form between accumulated cases for *Plasmodium falciparum* in a given transmission season and mean temperatures in a critical window of time that corresponds to the rainy season preceding transmission. For detailed description of these seasonal patterns, see 7. There are two transmission seasons each year: the first one from May to August, the second one from September to December. The corresponding rainy seasons are respectively from February to May and from June to September. (A) and (B) Time series of mean monthly temperatures for the two rainy periods. (C) Boxplot showing the seasonality of monthly cases for *Plasmodium falciparum*. (D) and (E) Scatter plots of cumulated cases as a function of mean temperatures.

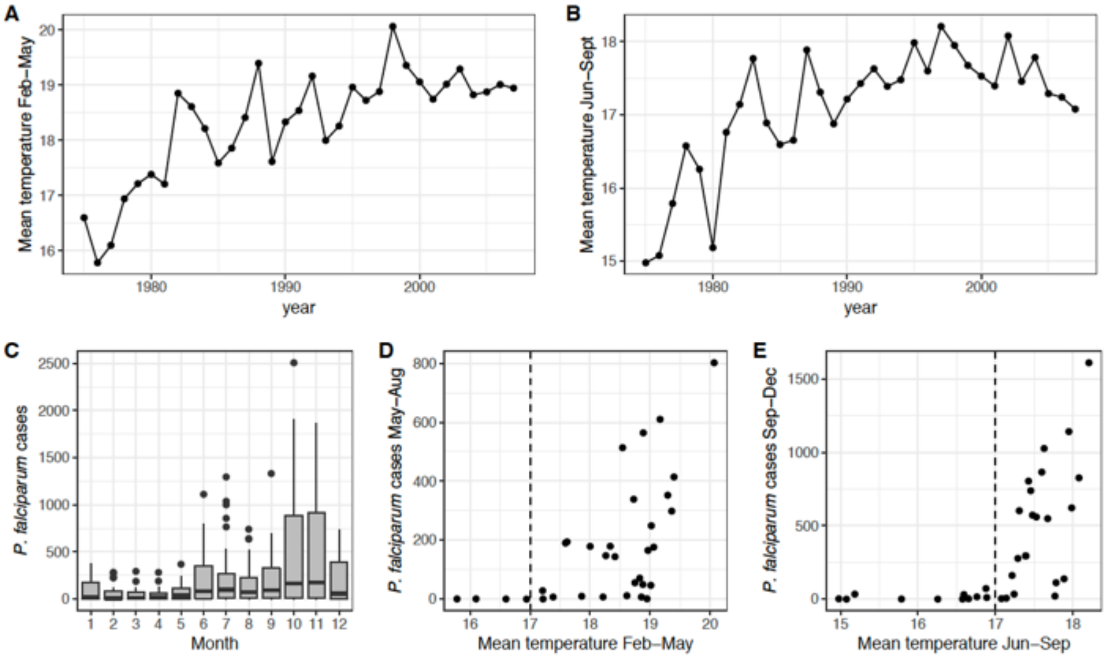

Supplementary Figure 5: Comparison of reported cases with predictions obtained with the transmission model whose incubation period is fixed at 12 days. The reported cases are shown in red. The median predicted cases are shown for the training set in blue, and for the 'out-of-fit' set in green, as in Figure 3 of the main text with the corresponding uncertainty shaded (for the 10% and 90% quantiles). Results are similar to those for the model where this parameter was estimated.

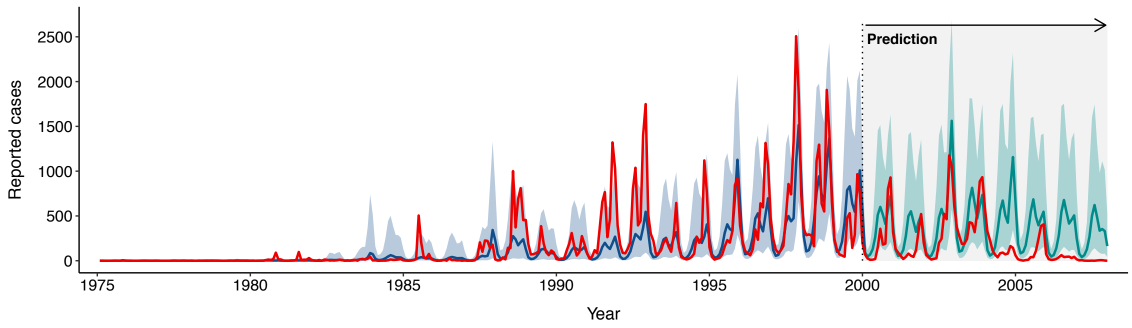

Supplementary Figure 6: Estimated transmission rate and is interannual variation as a function of temperatures. (A) The b-splines used to fit the seasonality of the transmission rate $\beta(t)$. Two splines (s_4 and s_6) are emphasized in bold lines. These are the splines used to anchor the temporal location of the interannual effect of temperature via the two respective covariates $TEMP_1$ and $TEMP_2$ (see expression of $\beta(t)$ in Methods). (B) The estimated transmission rate shown as a function of month for different years. Note the increase transmission from 1975 to 1997 and 1998, and the high transmission rate in 1997/98 coincident with the ENSO event of that period.

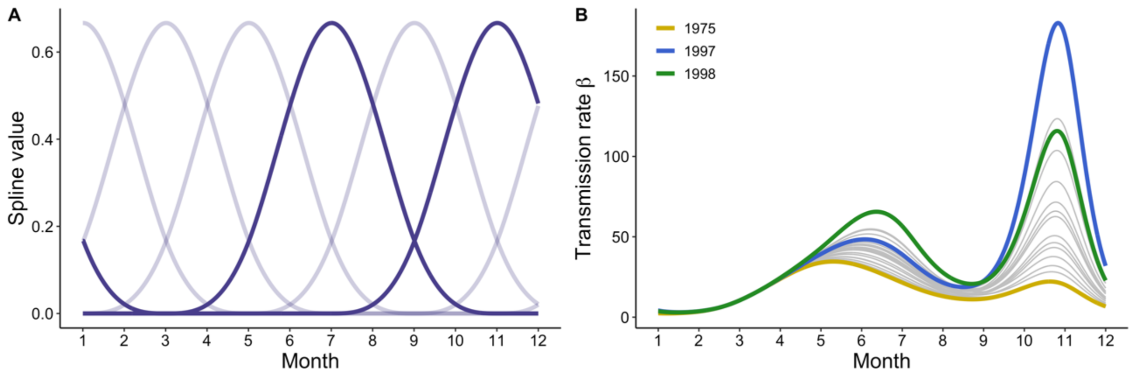

Supplementary Figure 7: Comparison of observed falciparum malaria cases and predicted values with the transmission model driven by minimum temperatures. In this model, the temperature covariates $TEMP_1$ and $TEMP_2$ are defined as a function of minimum temperature anomalies (see suppl. Fig. 8 for details). The reported cases are shown in red. Median simulated cases (hindcasts) with the best model for the MLE parameters are shown in blue for the time period of the training set data together with their uncertainty (shaded, for the 10% and 90% quantiles). Median predictions for the 'out-of-fit' period are shown in green, also with their corresponding uncertainty.

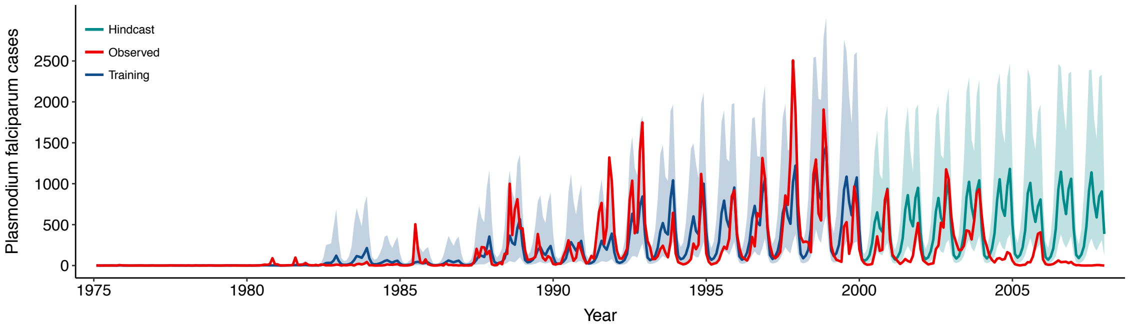

Supplementary Figure 8: Definition of temperature covariates $TEMP_1$ and $TEMP_2$ on the basis of minimum temperature in alternative transmission model. Top row: Mean monthly minimum temperature anomaly as a function of year for the same time windows used in the main model (February-May and June-September), which correspond respectively to the rainy season preceding each of the two malaria seasons (see Methods). Bottom row: Aggregated cases for the malaria season that follows each of these intervals plotted as a function of minimum temperature anomalies. On the basis of these relationships, we defined $TEMP_1$ and $TEMP_2$ in this model as these mean anomalies in minimum temperature for each of the time windows respectively.

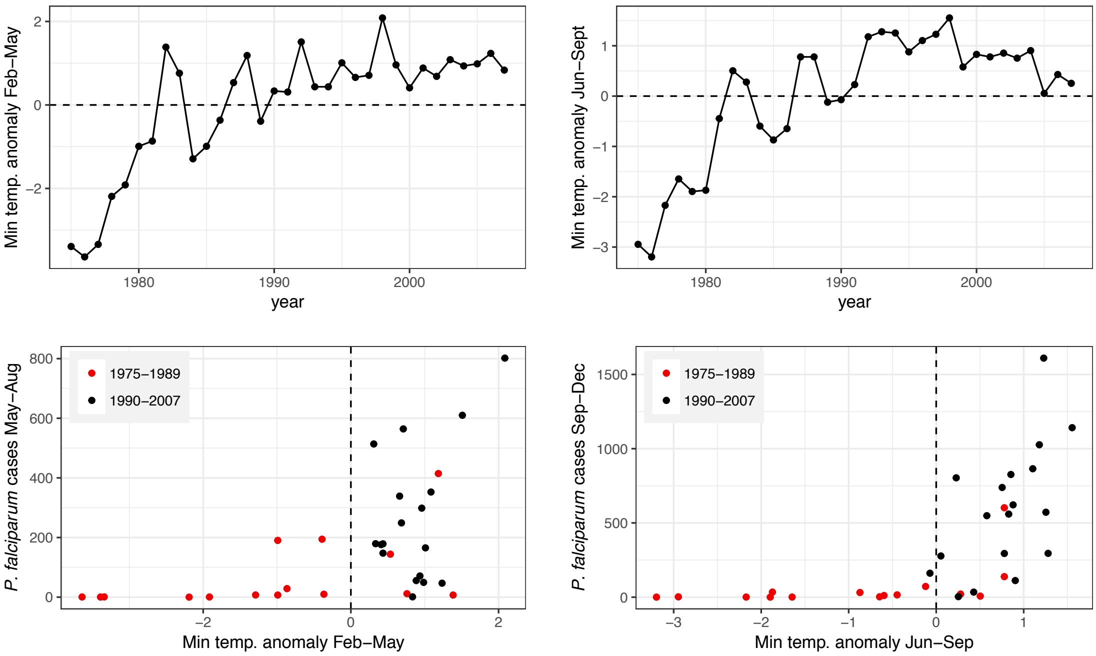

Supplementary Figure 9: Temporal SDC analysis between the time series of Niño3.4 and P_f cases. As for the application to SDC maps, this analysis seeks to describe associations that are transitory in time; thus, correlations are computed repeatedly for a restricted temporal window that moves along the two time series. Here that window corresponds to the main EN periodicity of 3.6-yr, or 43 months. Results are displayed as follows: (1) the two time series, respectively, to the left and top of the matrix of correlation values; (2) the matrix itself in the central panel with positive correlations in orange and negative ones in blue, with rows and columns corresponding to the temporal localization of the moving window for the time series on the left and top respectively; (3) the time series at the bottom of the highest and significant correlations for a given time (vertically, and therefore, for the variable that acts as the driver, here Niño3.4. To read the results, one starts at the diagonal and moves vertically off from it to identify a given lag for which significant correlations are found (the closest to the main diagonal). Here, time intervals with high local correlations are highlighted with the black circles (for EN86-LN87 and EN97-LN99). These intervals alternate with another encompassing other EN events for which no significant correlation is found, indicated with the red circle (for the 1991-94 EN interval). All colored areas depict significance levels of at least $p<0.05$. SDC has been specifically developed to test for significance of correlations in the local windows of time using permutations (see Rodó and Rodríguez-Arias, 2006 for details). The colorbar indicates correlation values.

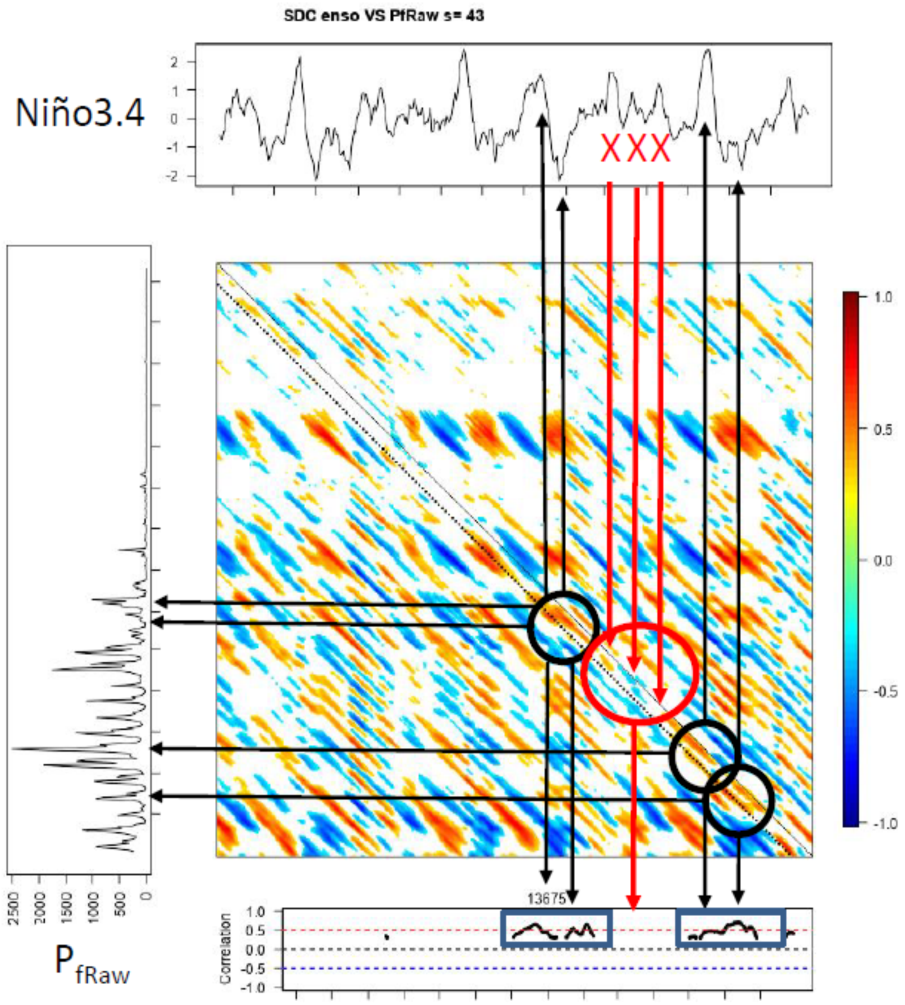

Supplementary Figure 10: SDC Maps between P_f and surface air temperature (T_{2m}). Correlations are shown for the malaria time series in a localized time window coinciding with the strongest EN event (1997/98) and T_{2m} in a global grid, for comparison with Figure 5 (where global SST anomalies where considered instead). The panels correspond to different lags between the climate variable and the malaria peak, namely with T_{2m} leading by 4 months in (A) and 2 months in (B), exhibiting no lag in (C), and lagging by 2 months in (D). See other details in the caption of that figure.

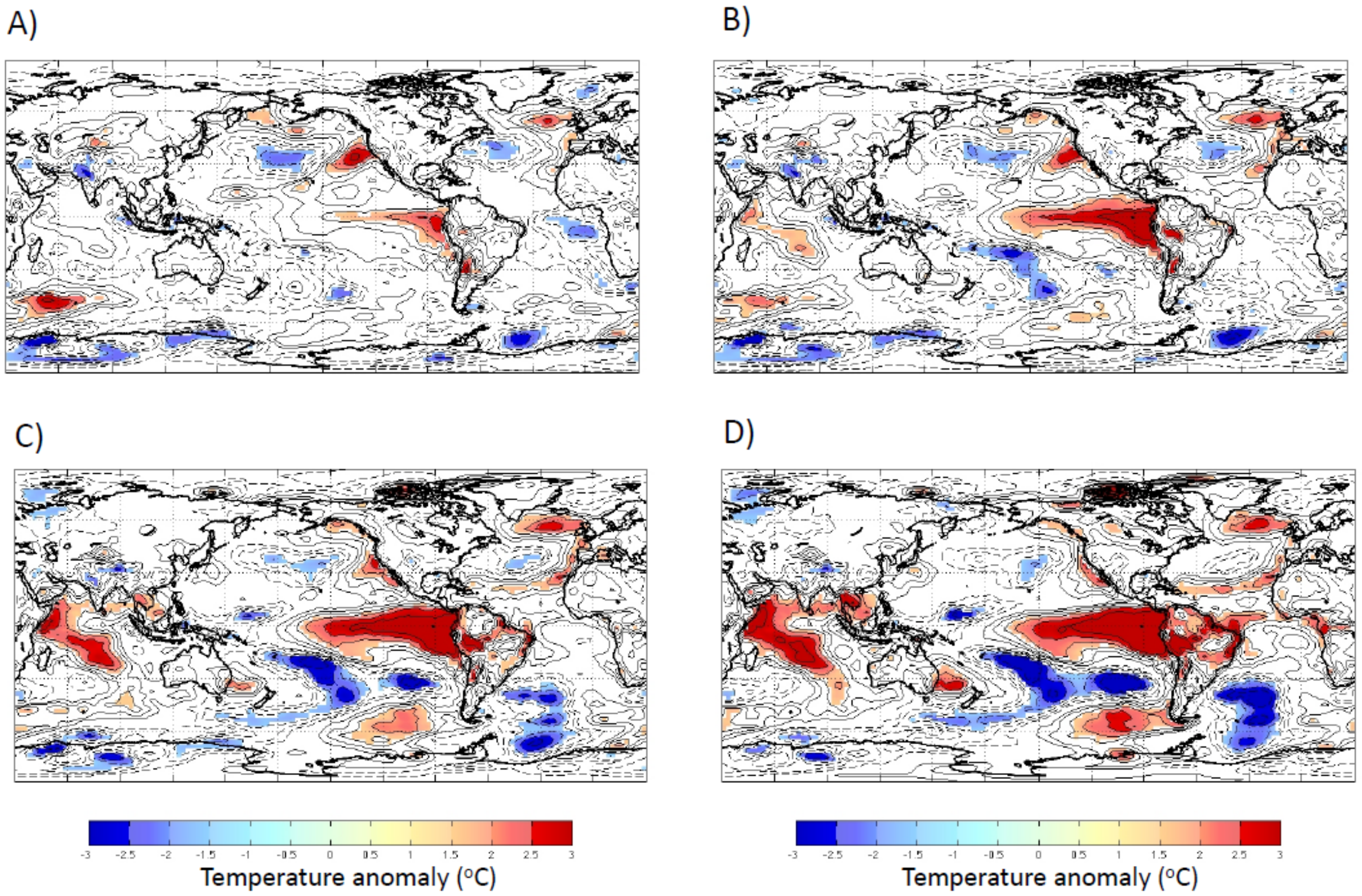
A)
B)
C)
D)
-3 -2.5 -2 -1.5 -1 -0.5 0 0.5 1 1.5 2 2.5 3
Temperature anomaly (°C)
-3 -2.5 -2 -1.5 -1 -0.5 0 0.5 1 1.5 2 2.5 3
Temperature anomaly (°C)

Supplementary Figure 11: **SDC Maps between the Niño3.4 index and the global grid of SST anomalies** (as for Fig. 5). The three top rows contain for each panel the analysis of an individual EN event. That is, the window of time for which the correlation is computed is placed over the specific event (and spans 24 months for both time series). The lag between the two time series is 0. The events are ordered from top to bottom and from left to right, by the decreasing order of magnitude of the corresponding size of the anomaly in the Niño3.4 time series. (That is, the top-left plot corresponds to the 1997/98 EN, the second one from there, to the 1981/82 EN, the third one, to the 1986/87 EN, and so on, for the years indicated on top of each panel). The last panel depicts the ensemble average for the 11 events.

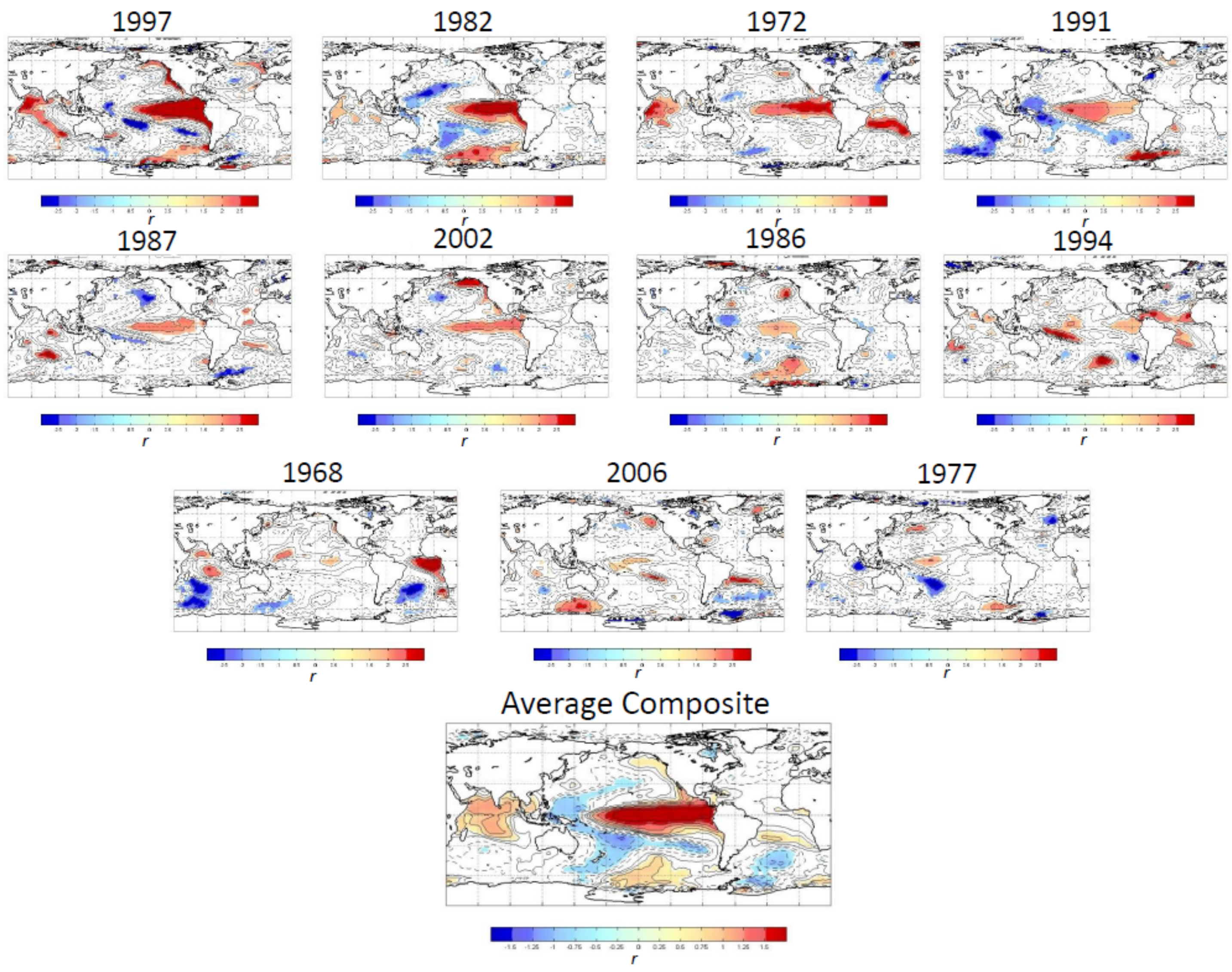
1997
1982
1972
1991
1987
2002
1986
1994
1968
2006
1977
Average Composite
r

Supplementary Figure 12: Time series and power spectra for the Indian Dipole Mode (IDM) and the ENSO index Niño3.4. A) Time series are shown from 1968 to 2008. The data represented are anomalies with regard to the long-term mean. B) Multi-taper Method (MTM) spectral analysis of the Niño3.4 index in A). C) Idem as B) but for the IDM index. Significance levels at 1% and 5% are denoted by horizontal lines.

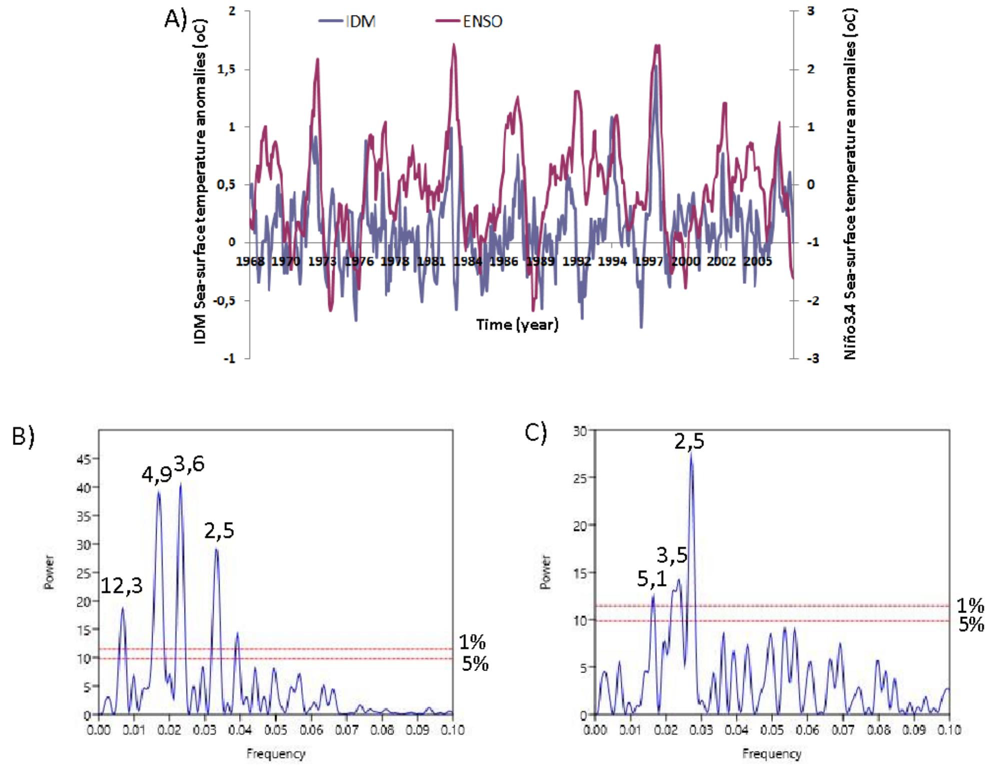

Supplementary Figure 13: Trend in global mean surface temperature (GMST) and comparison of detrended GSMT and anomalies in ENSO and PDO. A) SSA decomposition of the global mean surface temperature (HadCRTU4, blue), with its trend (HadCRTU4 TR) for the interval 1968-2005 superimposed. The trend TR corresponds to the first reconstructed component (RC) obtained after applying a SSA decomposition with an embedding dimension M=100 (see Methods). B) Time series of the detrended GMST series in A (HadCRTU4 no TR, blue) and the PDO (red) and, C) GMST series in A (HadCRTU4 no TR, blue) and the Niño 3.4 (red).

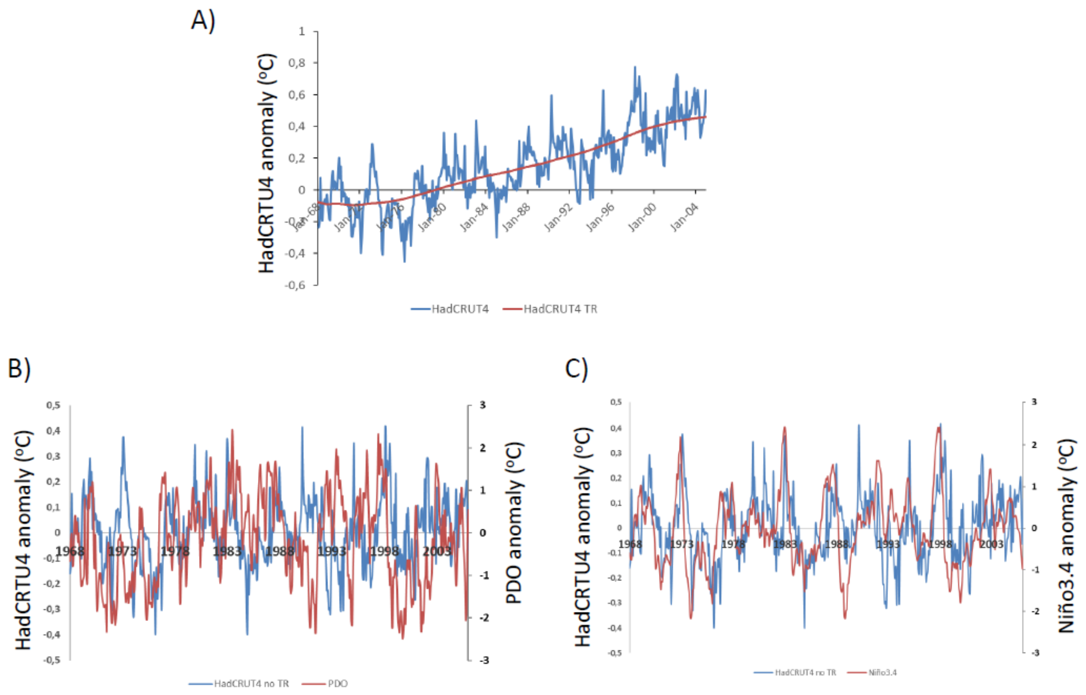

Supplementary Figure 14: Correlations of the Niño3.4 index with simulated sea and land surface temperatures in two time intervals, before A) 1979-2000 and, after B) 2001-2016, the slowdown, respectively. Ensemble mean simulations (10 members) are shown for the consensus atmospheric model simulations for the region of interest with the Community Atmosphere Model version 5 (CAM5[42]). (see Methods). (http://www.cesm.ucar.edu/models/cesm1.0/cam/docs/description/cam5_desc.pdf). Values over 0.226 in absolute correlation denote 5% significance level.

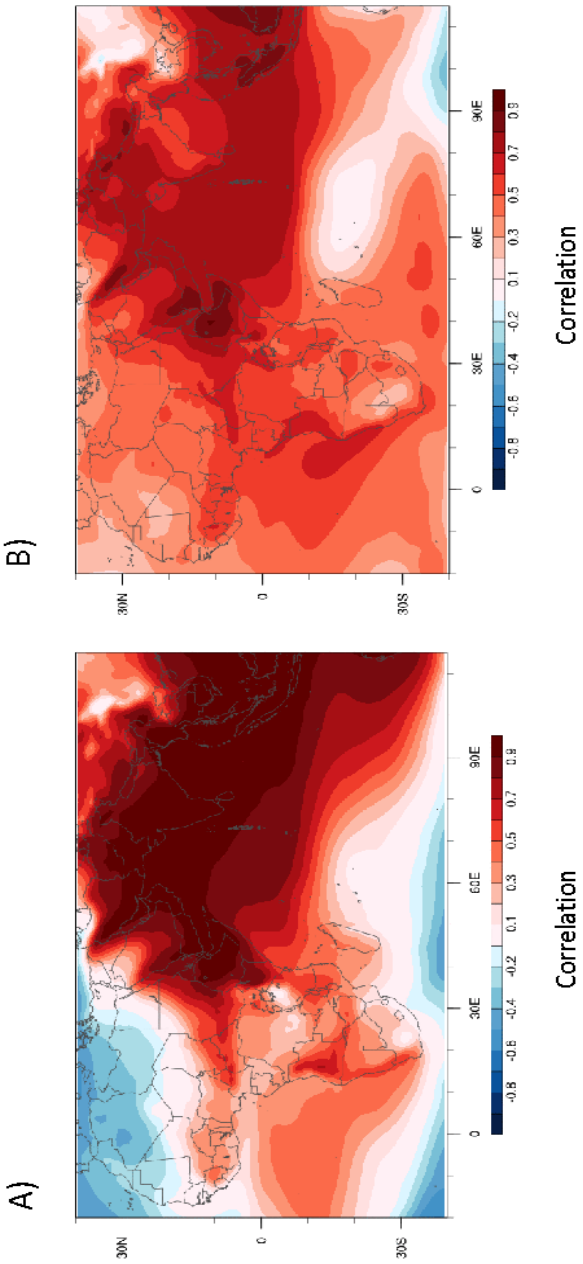

Supplementary Figure 15: Correlations of the PDO with simulated sea and land surface temperatures in two time intervals, before A) 1979-2000 and, after B) 2001-2016, the slowdown, respectively As Suppl. Fig. 12 but for spatial correlations with the PDO.

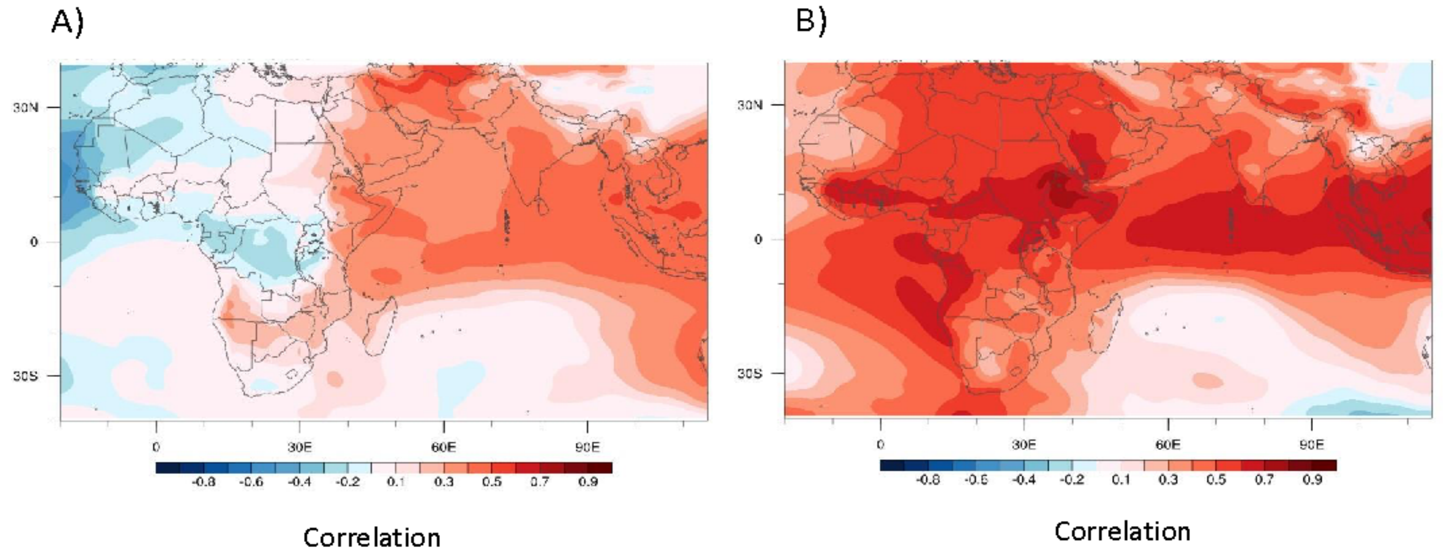
A)
30N
0
30S
0
30E
60E
90E
-0.8
-0.6
-0.4
-0.2
0.1
0.3
0.5
0.7
0.9
Correlation
B)
30N
0
30S
0
30E
60E
90E
-0.8
-0.6
-0.4
-0.2
0.1
0.3
0.5
0.7
0.9
Correlation

Supplementary Table I: Correlation coefficients for pairs of time series (reconstructed components) shown in Figure 3. Both significance levels with a randomization test accounting for the decrease in the d.o.f. and the lag at which the maximum correlation is attained are both shown in columns, together with the location (panels A,B or C in Fig. 3). “LT” and “ann” refer to long-term and annual components, respectively.

Variables	Correlation coef.	p-value	Lag	Figure in manuscript
T_{min}, P_v (LT)	0.986	<0.01	0	3A
T_{min}, P_f (LT)	0.978	<0.01	0	3A
P_f, P_v (LT)	0.981	<0.01	0	3A
T_{min}, R (ann)	0.724	<0.01	1	3B
P_f, P_v (ann)	0.956	<0.01	0	3C
