# Supplementary material for: Malaria trends in Ethiopian highlands track the 2000 ‘slowdown’ in global warming
Source: Nat Commun. 2021 Mar 10;12:1555. doi: 10.1038/s41467-021-21815-y (PMC7946882; doi:10.1038/s41467-021-21815-y)
Supplement: Supplementary file 3 — Reporting Summary [file 41467_2021_21815_MOESM3_ESM.pdf]

nature research

Double-blind peer review submissions: write DBPR and your manuscript number here instead of author names.

Corresponding author(s): Mercedes Pascual

Last updated by author(s): 2021-01-25 M. Pascual et al.

Reporting Summary

Nature Research wishes to improve the reproducibility of the work that we publish. This form provides structure for consistency and transparency in reporting. For further information on Nature Research policies, see our Editorial Policies and the Editorial Policy Checklist.

Statistics

For all statistical analyses, confirm that the following items are present in the figure legend, table legend, main text, or Methods section.

n/a	Confirmed	
☒	☐	The exact sample size (*n*) for each experimental group/condition, given as a discrete number and unit of measurement
☒	☐	A statement on whether measurements were taken from distinct samples or whether the same sample was measured repeatedly
☐	☒	The statistical test(s) used AND whether they are one- or two-sided<br>*Only common tests should be described solely by name; describe more complex techniques in the Methods section.*
☐	☒	A description of all covariates tested
☒	☐	A description of any assumptions or corrections, such as tests of normality and adjustment for multiple comparisons
☐	☒	A full description of the statistical parameters including central tendency (e.g. means) or other basic estimates (e.g. regression coefficient) AND variation (e.g. standard deviation) or associated estimates of uncertainty (e.g. confidence intervals)
☐	☒	For null hypothesis testing, the test statistic (e.g. *F*, *t*, *r*) with confidence intervals, effect sizes, degrees of freedom and *P* value noted<br>*Give P values as exact values whenever suitable.*
☒	☐	For Bayesian analysis, information on the choice of priors and Markov chain Monte Carlo settings
☒	☐	For hierarchical and complex designs, identification of the appropriate level for tests and full reporting of outcomes
☒	☐	Estimates of effect sizes (e.g. Cohen's *d*, Pearson's *r*), indicating how they were calculated

Our web collection on statistics for biologists contains articles on many of the points above.

Software and code

Policy information about availability of computer code

Data collection	N/A . No software was used for data collection
Data analysis	For fitting the transmission model and implementing predictions with the resulting model, we used the R Package pomp (for Statistical Inference for Partially Observed Markov Processes via) and in particular, the function MIF for iterated particle filtering. For Scale Dependent Correlation (SDC) we use routines developed by one of the authors (X.R.) in both R and Python. We have made available the codes for pomp and SDC in respective Github repositories (see links in Code Availability statement). For Singular Spectrum Analysis (SSA) and associated MTM, we used the UCLA SSA Toolkit version 4.4 (https://web.atmos.ucla.edu/tcd/ssa/), and the spectrum.mtm script within Matlab (Matlab_R2019a).

For manuscripts utilizing custom algorithms or software that are central to the research but not yet described in published literature, software must be made available to editors and reviewers. We strongly encourage code deposition in a community repository (e.g. GitHub). See the Nature Research guidelines for submitting code & software for further information.

Data

Policy information about availability of data

All manuscripts must include a data availability statement. This statement should provide the following information, where applicable:
- Accession codes, unique identifiers, or web links for publicly available datasets
- A list of figures that have associated raw data
- A description of any restrictions on data availability

'The AC&C/SPARC ozone database as well as the other input data for the atmospheric simulations are part of the customized initial and boundary conditions used in the simulations specified in the Methods, and are all accessible from the following link:
http://www.pa.op.dlr.de/CCMVal/AC&CSPARC_O3Database_CMIP5.html
Monthly averages of daily minimum and maximum temperature and precipitation for stations of the Oromia region were obtained from the Ethiopian National Meteorological Agency (NMA). These data can be found at https://github.com/pascualgroup/Malaria-highlands. The El Niño index (Niño 3.4) and PDO index were obtained from www.esrl.noaa.gov/psd/. The reanalysis data can be found at https://crudata.uea.ac.uk/cru/data/hrg/.
The epidemiological data are available from the authors upon request.

Figure 1 includes raw data.

Field-specific reporting

Please select the one below that is the best fit for your research. If you are not sure, read the appropriate sections before making your selection.

☐ Life sciences ☐ Behavioural & social sciences ☒ Ecological, evolutionary & environmental sciences

For a reference copy of the document with all sections, see nature.com/documents/nr-reporting-summary-flat.pdf

Ecological, evolutionary & environmental sciences study design

All studies must disclose on these points even when the disclosure is negative.

Study description
We address the link between the reversal in malaria's decadal trend and the concomitant temporary slowdown in global warming by considering its regional manifestation in local temperatures, as well as the role of ENSO and the PDO on regional climate. We take advantage of extensive retrospective records on malaria cases for both parasites Plasmodium falciparum (Pf) and Plasmodium vivax (Pv) with different epidemiology in a highland region of Oromia, Ethiopia, where intense public health intervention did not start until 2005. We also analyze the full ensemble of weather stations for this region. In addition to variance and trend decompositions of local malaria, rainfall and temperature time series, we examine connections between malaria cases, regional climate and global climate variability. These connections help establish a consistent chain of effects and assess consistency of mechanisms and their associated variability across scales. In particular, a process-based transmission model is used to predict what would have been the effect on falciparum malaria cases of the observed change in temperatures in the absence of the public health interventions introduced post-2004.

Research sample
The research sample consists of monthly malaria cases for Plasmodium falciparum and for Plasmodium vivax from 1968 to 2007.
Reported cases for both parasites were confirmed through microscopy examination of blood slides from febrile cases seeking diagnosis and treatment. The malaria data were provided by the government malaria center in Debre Zeit. Climate data sets are also monthly time series from local meteorological stations, data sets from reanalyses whose source is described in the Methods' section of the manuscript, and the output of global climate models whose source is also described in the manuscript.

Sampling strategy
We relied on existing data sets from passive surveillance efforts. A calculation of sampling size does not apply and was not performed.

Data collection
We did not collect the data ourselves. The malaria data was obtained from the passive surveillance conducted at clinics and blood samples analyzed as part of this surveillance through microscopy to determine the presence of the parasites. This is standard practice.

Timing and spatial scale
The temporal frequency of the data sets we used is monthly and the epidemiological time series covers from January 1968 to December 2007. The spatial scale corresponds to the aggregation of 159 kebeles, the smallest administrative unit in Ethiopia, with an approximate total area of about 3500 km2.

Data exclusions
For consistency, cases diagnosed in government clinics outside the original reporting sector between 1993 and 2007 were not included.

Reproducibility
The analyses are reproducible on the basis of the information and codes provided.

Randomization
This is not relevant to our study as we relied on time series data from epidemiological surveillance by the public health system in Ethiopia, and on climate time series from climate data sets and global climate simulations.

Blinding
Blinding is not applicable as this is not a trial. The data for the study are longitudinal records from passive surveillance and climate datas sets

Did the study involve field work? ☐ Yes ☒ No

Reporting for specific materials, systems and methods

We require information from authors about some types of materials, experimental systems and methods used in many studies. Here, indicate whether each material, system or method listed is relevant to your study. If you are not sure if a list item applies to your research, read the appropriate section before selecting a response.

Materials & experimental systems

n/a	Involved in the study	
☒	☐	Antibodies
☒	☐	Eukaryotic cell lines
☒	☐	Palaeontology and archaeology
☒	☐	Animals and other organisms
☒	☐	Human research participants
☒	☐	Clinical data
☒	☐	Dual use research of concern

Methods

n/a	Involved in the study	
☒	☐	ChIP-seq
☒	☐	Flow cytometry
☒	☐	MRI-based neuroimaging
